# Correlation of invasive and non-invasive blood pressure: A must for management

**DOI:** 10.4103/0019-5049.72658

**Published:** 2010

**Authors:** Sameer Sethi

**Affiliations:** Department of Anaesthesia and Intensive Care, Post Graduate Institute of Medical Education and Research, Chandigarh, India

Sir,

Arterial blood pressure (ABP) is a basic haemodynamic index often utilized to guide therapeutic interventions, especially in critically ill patients. Inaccurate ABP measuring creates a potential for misdiagnosis and mismanagement. I would like to share an important and interesting experience with you that would be valuable for the students.

A 74-year-old male (43 kg, ASA status II)) patient was posted for parotid gland excision. The patient’s medical history was significant for chronic obstructive lung disease. He had fair effort tolerance (New York Heart Association-II). All pre-operative investigations were normal except for moderate obstruction in spirometry and typical hyperinflation features in chest X-ray. His physical examination and airway assessment were unremarkable.

The patient was induced with i.v. inj fentanyl 2 ug/kg inj propofol 2 mg/kg and inj atracurium 0.5 mg/kg after pre-oxygenating with 100% oxygen. His non-invasive systolic blood pressure was between 80 and 90 mmHg. We placed a radial arterial cannula with 100 cm of stiff tubing that was free of air bubbles and used a Flotrac™ sensor for arterial pressure monitoring. Invasive BP was 176/114 mmHg. There was no ringing or resonance and arterial tracing was absolutely normal. Non-invasive BP showed 82/46 mmHg. We cross-checked our BP reading and found it to be correct. Then, we changed the disposable transducer. After changing the disposable transducer, invasive BP correlated with non-invasive BP. The remaining intra-operative period was uneventful and the patient was extubated successfully.

**Figure 1:**
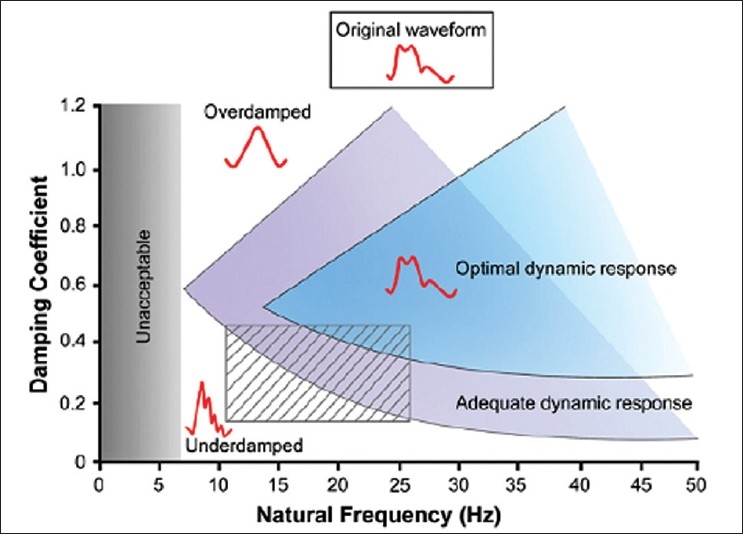
Relationship between damping and natural frequency of pressure monitoring systems, describing the characteristics of typical transducer systems in clinical use

Ideally, the pressure waves recorded through the intravascular catheter should be transmitted undistorted to the transducer and then to the amplifier, display or recording system.[1,2] Unfortunately, the mechanical transmission system oscillates (rings or resonates) after being set in motion by the arterial pressure wave. These oscillations produce small pressure waves that are superimposed on those caused by the pressure pulse itself, thereby introducing artefacts that distort the measured pressure.[1,3] In our case, there was no ringing or resonance as the arterial waveform was not distorted. Most disposable transducers have natural frequencies of several hundred hertz, but the addition of saline-filled tubing and stopcocks that may trap tiny air bubbles results in a monitoring system with a markedly reduced natural frequency.[1,4] We used 100 cm of stiff tubing that was free of air bubbles and a Flotrac™ sensor for arterial pressure monitoring. In our case, the disposable transducer was faulty, probably having a natural frequency in the unacceptable low range [Figure 1].[1] Most monitoring systems are underdamped but have a natural frequency high enough such that the effect on the monitored waveform is limited.[2]

In conclusion, invasive BP should always be correlated with non-invasive BP, and any discrepancy noted should be rectified to avoid misdiagnosis and mismanagement.
